# The Effect of Acupuncture on Glucose Metabolism and Lipid Profiles in Patients with PCOS: A Systematic Review and Meta-Analysis of Randomized Controlled Trials

**DOI:** 10.1155/2021/5555028

**Published:** 2021-03-22

**Authors:** Ruqun Zheng, Peng Qing, Mei Han, Jinlong Song, Min Hu, Hongxia Ma, Juan Li

**Affiliations:** ^1^Department of Traditional Chinese Medicine, The First Affiliated Hospital of Guangzhou Medical University, Guangzhou, China; ^2^Department of Acumoxibustion, The First Affiliated Hospital of Jinan University, Guangzhou, China; ^3^Beijing University of Chinese Medicine, Beijing, China; ^4^Department of Laboratory, The First Affiliated Hospital of Guangzhou Medical University, Guangzhou, China

## Abstract

**Objective:**

To evaluate the effectiveness of acupuncture on glucose metabolism and lipid profiles in patients with polycystic ovary syndrome (PCOS).

**Methods:**

Databases, including the China National Knowledge Infrastructure (CNKI), the China Science and Technology Journal Database (VIP), Wanfang, PubMed, and the Cochrane Library were searched for the relevant literature, with the retrieval deadline being February 2020. Two reviewers independently screened, selected, and extracted the data and validated the results. The methodological quality of the included studies was evaluated with the risk of bias tool, and the meta-analysis was performed using the RevMan 5.3.5 software.

**Results:**

A total of 737 patients with PCOS from 10 randomized controlled trials were included in the meta-analysis. A pooled analysis showed significant decreases in body mass index (mean difference (MD) = –1.47, 95% CI –2.35 to –0.58, *P* < 0.001) and waist-to-hip ratio (MD = –0.04, 95% CI [–0.06, –0.02], *P* < 0.001) in the acupuncture group along with significant improvements in fasting plasma glucose (MD = –0.38, 95% CI [–0.70, –0.07], *P* = 0.02), homeostasis model assessment of insulin resistance (MD = –0.22, 95% CI [–0.41, –0.02], *P* = 0.03), and triglycerides (MD = –0.26, 95% CI [–0.48, –0.04], *P* = 0.02). No significant differences were observed in the Ferriman–Gallwey score, 2 h fasting plasma glucose, fasting insulin, 2 h fasting insulin, serum total cholesterol, low-density lipoprotein cholesterol, or high-density lipoprotein cholesterol.

**Conclusion:**

Acupuncture is relatively effective and safe in improving glucose metabolism and insulin sensitivity in patients with PCOS. The included studies were generally of not bad methodological quality, but further large-scale, long-term randomized controlled trials with rigorous methodological standards are still warranted.

## 1. Introduction

Polycystic ovary syndrome (PCOS) is a complex endocrine disorder in reproductive-age women with an incidence of 6% to 21% globally and 5.6% to 11.2% in Chinese women [1]. PCOS generally manifests as hyperandrogenism, oligoanovulation, and/or polycystic ovaries [2], and patients with PCOS are at an increased risk for metabolic disturbances such as insulin resistance (IR), impaired glucose tolerance (IGT), dyslipidemia, insulin-induced metabolic syndrome predisposing them to type 2 diabetes mellitus (T2D), and cardiovascular disease [3–5]. Furthermore, hyperinsulinemia is considered to be associated with the mechanism of ovulatory dysfunction in women with PCOS [6].

IGT, obesity, and T2D are more prevalent in women with PCOS women than in the general population [6–8], and it is estimated that among obese women with PCOS about 30%–40% are diagnosed with IGT and 5–10% suffer from T2D [9, 10]. Moreover, metabolic syndrome is three times more frequent in women who present with classic PCOS symptoms [11]. The metabolic disturbances present in women with PCOS not only involve an increased risk for cardiovascular disease but also might worsen many of the typical PCOS symptoms, and thus they have become an important target for therapy [12].

Lifestyle measures, including diet, exercise, and behavioral modification, are the first-line treatments in obese women with PCOS for improving metabolic parameters and endocrine abnormalities [13]. Nevertheless, lifestyle changes might not be easy to sustain and might not be sufficient for significant weight loss. Thus, pharmaceutical methods targeting both reproductive and metabolic disorders might be needed.

Biguanides (metformin) and thiazolidinediones (rosiglitazone and pioglitazone) are insulin-sensitizing agents that are currently in clinical use [8]. Metformin has a few safety concerns and is the most widely used insulin-sensitizing agent for treating women with PCOS of reproductive age [6, 14]. Metformin is an oral antihyperglycemic biguanide drug that enhances glucose uptake in skeletal muscle and adipocytes by increasing insulin sensitivity, and it is often used as a first-line pharmacological treatment for women with PCOS presenting with IR [15–17]. However, gastrointestinal symptoms like diarrhea and abdominal discomfort are common adverse effects of metformin [18, 19]. In addition, rosiglitazone has been withdrawn from the market in many countries due to concerns of increased risk of congestive heart failure [20].

Pharmacological approaches are often effective, but there are issues with adverse effects and patient compliance due to the prolonged treatment required by PCOS patients [19]. Thus, additional nonpharmacological treatment strategies such as acupuncture should be considered in treating PCOS [19]. Acupuncture, which has been used to treat diseases for more than 2500 years in China [21], has become increasingly popular worldwide in recent years for its convenience and low incidence of adverse effects. Acupuncture is widely applied in clinical practice for treating PCOS [19], and studies have concluded that acupuncture is effective against metabolic disturbances associated with IR such as overweight [22], hyperglycemia [23, 24], and hyperlipidemia [25] by improving insulin sensitivity [26]. It has also been reported that acupuncture may improve glycemic outcomes in women with PCOS [27]. Our prospective pilot study [28] showed that homeostasis model assessment of insulin resistance (HOMA-IR) was decreased after 6 months of treatment with acupuncture, and HOMA-IR remained significantly decreased at 3 months of follow-up. In addition, electroacupuncture has been shown to decrease HOMA-IR and improve IR in a rat model of PCOS [29]. However, the designs of these studies were all considerably different and thus it is difficult to draw strong conclusions, and the effects of acupuncture on glucose and lipid metabolism in PCOS have not been systematically analyzed.

To fill this knowledge gap, this systematic review aimed at comparing the effectiveness of acupuncture to that of standard therapy (lifestyle management or insulin-sensitizing agents) in the treatment of metabolic dysfunction in patients with PCOS.

## 2. Methods and Materials

### 2.1. Eligibility Criteria

We only included randomized controlled trials (RCTs) using accepted interventions involving acupuncture (manual acupuncture or electroacupuncture) alone, with unrestricted acupoints or intensity, compared with placebo (placebo or sham acupuncture) or with standard therapy (lifestyle management, including weight reduction by diet and exercise or insulin-sensitizing agents such as thiazolidinediones and metformin). The subjects were adult patients with PCOS. Only studies with the outcomes of HOMA-IR, HbA1c, glucose, and insulin levels or lipid profiles were included.

### 2.2. Literature Search

We searched for published literature in the China National Knowledge Infrastructure (CNKI), Wanfang, the China Science and Technology Journal Database (VIP), PubMed, and the Cochrane Library databases. We also retrieved the completed but unpublished studies from the clinicaltrials.gov website and tracked the results of these studies. Only Chinese and English articles were retrieved, and the last search was carried out on 29 February 2020. The search words included “polycystic ovary syndrome”, “PCOS”, “acupuncture”, “electroacupuncture”, “needle”, “needling”, “scalp acupuncture”, “abdominal acupuncture”, “ear acupuncture”, “wrist-ankle acupuncture”, “warm acupuncture-moxibustion”, “homeostatic model assessment”, “HOMA”, “glucose”, “insulin”, “insulin sensitivity”, “metabolic”, “glycemic control”, “OGTT”, “lipid profile”, “HbA1c”, “triglycerides (TG)”, “total cholesterol (TC)”, “high-density lipoprotein cholesterol (HDL-C)”, “low-density lipoprotein cholesterol (LDL-C)”, “randomized controlled trial”, “clinical trial”, “RCT”, “random”, “randomize”, and “randomization”. Depending on the characteristics of different databases, search strategies including both subject words + free words and keywords + full text were used.

### 2.3. Literature Screening

The identified articles were initially imported into NoteExpress, and the initial screening was performed based on the inclusion/exclusion criteria after reading the titles and abstracts. In the next step, full-text articles were acquired and checked for eligibility prior to including them in the final analysis. All duplicated articles and any papers that did not meet our inclusion criteria were excluded.

### 2.4. Data Extraction

A data extraction table was designed to collect the data to be analyzed, including the following aspects: (a) basic characteristics of the included studies; (b) research methods and possible biases; (c) participant characteristics; (d) interventions; (e) outcome measures; (f) research findings; and (g) other required information.

Two investigators (Zheng and Qing) independently extracted the data and assessed the quality of all relevant RCTs. The following data were extracted from the published RCTs: the first author's name, year of publication, the country where the trial was conducted, the type of study, the intervention and placebo groups, the frequency of acupuncture or the dosage of pharmaceutical interventions (mg/day), the duration of intervention, the sample size, the mean and standard deviation of the glucose metabolism outcomes, and the mean and standard deviation of the lipid profile outcomes. Microsoft Excel with standard spreadsheets was used for data extraction.

### 2.5. Quality Evaluation

The risk of bias among the included studies was evaluated using the tool developed by the Cochrane Collaboration [30]. The results were cross-referenced, and any disagreements were resolved by discussion or consultation with a third evaluator with rich experience.

### 2.6. Outcome Measures

The primary outcome of interest was HOMA-IR. The secondary outcomes were fasting plasma glucose (FPG), fasting plasma insulin (FINS), 2 h fasting plasma glucose (2hFPG), 2 h fasting insulin (2hFINS), TC, TG, HDL-C, LDL-C, body mass index (BMI), waist-to-hip ratio (WHR), and the Ferriman–Gallwey score (FGS). The safety indicator was any adverse event.

### 2.7. Data Analysis

The quantitative analysis was carried out using the Cochrane Collaboration software RevMan 5.3.5. If the included studies used the same measurement scales, the continuous variables were described using the mean difference (MD) and 95% confidence intervals (CIs). For the heterogeneity test, the chi-square test was performed first, and based on those results, the estimates of heterogeneity (*I*^2^) were applied. A fixed-effect model was used when the *I*^2^ was ≤50% and the *P* value was ≥0.1, and a random-effect model was applied when the *I*^2^ was >50% or the *P* value was <0.10. If heterogeneity was high, the source of heterogeneity was explored, and subgroup analysis or sensitivity analysis was performed to investigate the stability of the meta-analysis.

## 3. Results

### 3.1. Literature Search and Screening Flowchart

**Figure 1:**
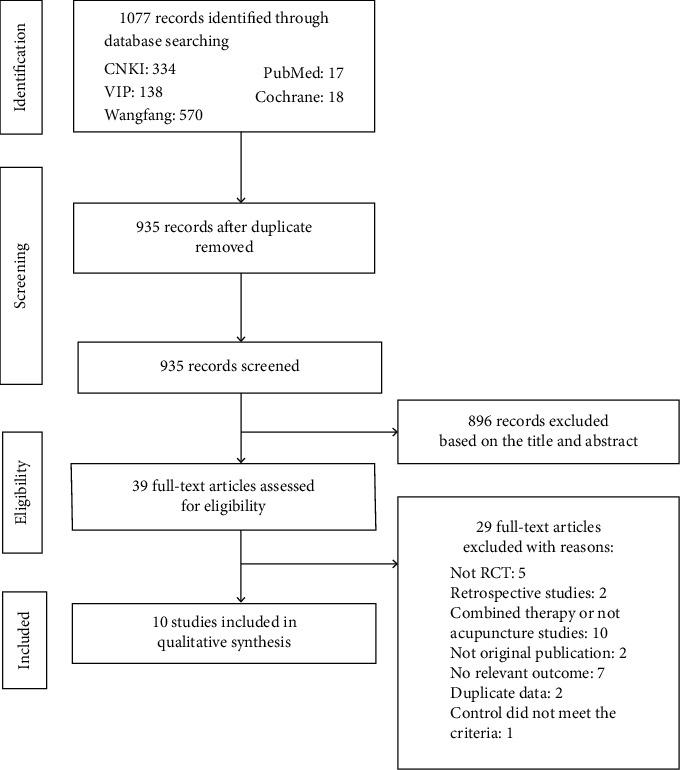
Literature search and screening flowchart.

A flow chart of the study selection is shown in Figure 1. In all, 1077 articles were retrieved in our initial search. After removing duplications and screening the titles and abstracts, we obtained 39 full-text articles. Finally, 10 studies were included in the systematic review and meta-analysis.

### 3.2. Characteristics of the Included Literature

A total of 737 patients were included in the 10 RCTs [31–40]. All participants were diagnosed with PCOS according to Rotterdam criteria [41], and they were treated with acupuncture alone or with placebo or metformin. The main acupoints used in these studies included CV3, RN4, RN6, ST25, ST28, ST36, SP9, and SP6. The specific features of these studies are summarized in Table 1.

### 3.3. Quality Evaluation of the Articles

**Figure 2:**
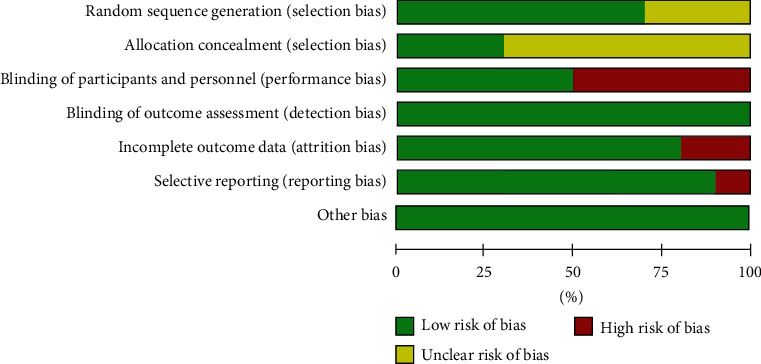
Evaluation of the risk biases of the included studies.

Among these 10 studies, the patients were randomized by using a random number table in six studies [31, 33–35, 37, 38], while the remaining four studies only mentioned “random” or “randomization” without describing the specific randomization methods. Only three articles described allocation concealment [31, 33, 40]. For participant and personnel blinding, five trials were at low risk of bias [31, 32, 38–40], and the remaining studies [33–37] were given a high risk of bias due to the loss of blinding during implementation. Measurements were generally made by third parties other than the researchers, so the blinding of outcome assessment was defined as low risk. Two articles failed to describe the missing data, while in the remaining eight articles, the number of patients in all randomized groups was consistent with the number of subjects in the statistical analysis. The articles reported both the glucose metabolism and lipid profile indicators. The quality of the literature included in our analysis was average, and the details of the evaluation are shown in Figure 2.

### 3.4. Results of the Meta-Analysis

#### 3.4.1. General Indicators

**Figure 3:**
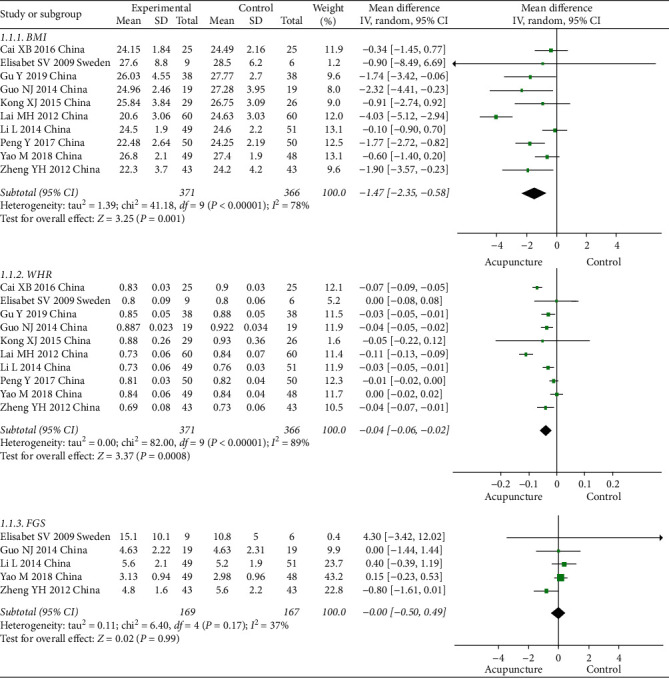
Comparison of the BMI, WHR, and FGS between the acupuncture and control groups in the treatment of PCOS.

All included studies compared BMI and WHR, while only five studies reported FGS. There was evidence for a decrease in BMI in the acupuncture groups versus the control groups (MD = –1.47, 95% CI [–2.35 to –0.58], *P* < 0.001). The overall effect showed a significant improvement in WHR between the groups (MD = –0.04, 95% CI [–0.06, –0.02], *P* < 0.001). No significant difference was observed in FGS. The results of the meta-analysis are shown in Figure 3.

#### 3.4.2. Glucose Metabolism Indicators

**Figure 4:**
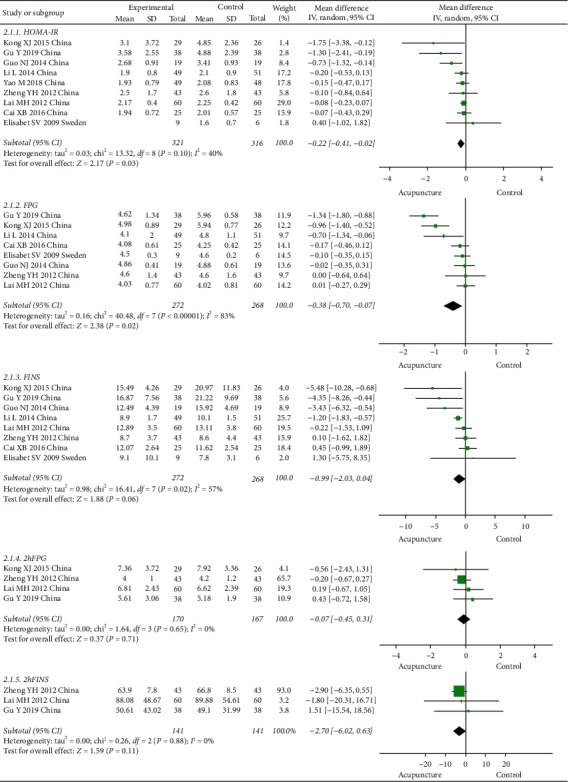
Comparison of glucose metabolism indicators between the acupuncture and control groups in the treatment of PCOS.

Nine articles reported HOMA-IR and eight articles reported FPG and FINS, but only four articles reported 2hFPG and only two reported 2hFINS. The pooled results from nine studies showed a significant difference in HOMA-IR in the acupuncture groups compared with the control groups (MD = –0.22, 95% CI [–0.41, –0.02], *P* = 0.03). The pooled analysis showed a decrease in FPG in the acupuncture group (MD = –0.38; 95% CI [–0.70, –0.07], *P* = 0.02). There was an improvement in FINS between the groups (MD = –0.99, 95% CI [–2.03, 0.04], *P* = 0.06), but this was not statistically significant. No significant differences were observed for the other outcomes. The results of the meta-analysis are shown in Figure 4.

#### 3.4.3. Lipid Profile

**Figure 5:**
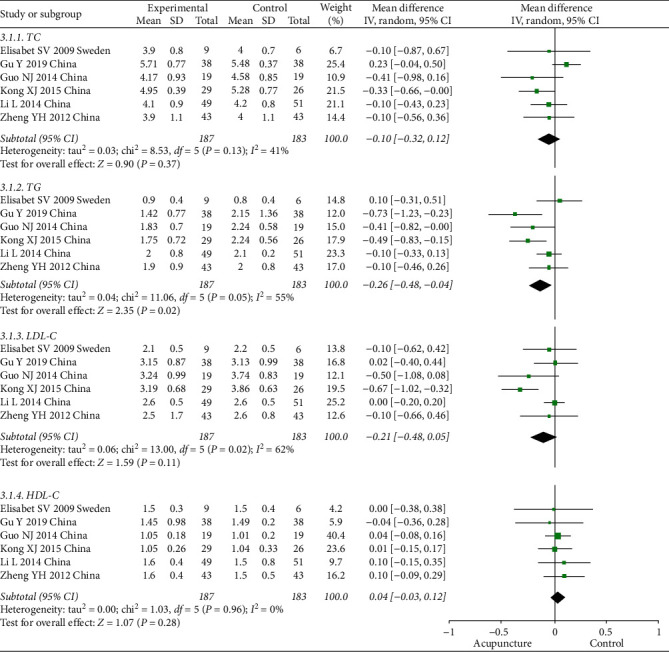
Comparison of lipid profiles between the acupuncture and control groups in the treatment of PCOS.

Six studies with 370 participants were included to compare TC, TG, LDL-C, and HDL-C between groups. There was no significant difference in TC or HDL-C between the acupuncture and control groups, but a significant decrease was observed in TG in the acupuncture group (MD = –0.26, 95% CI [–0.48, –0.04], *P* = 0.02). The results of the meta-analysis are shown in Figure 5.

#### 3.4.4. Adverse Events

Six of the studies reported the presence of adverse events. Of these, two studies [31, 40] reported that there were no adverse events, while the remaining four studies [33, 36–38] reported gastrointestinal problems in the metformin groups such as nausea, vomiting, mild diarrhea, slight dizziness, or weakness. There were no adverse events found in the acupuncture groups of these studies except for one study [37] in which one patient had mild bleeding at the site of needling.

## 4. Discussion

### 4.1. Principle Findings

The objective of this review was to summarize and evaluate the use of acupuncture to improve glucose metabolism and lipid profiles in patients with PCOS. Overall, we found that acupuncture was closely associated with decreased BMI, WHR, FPG, and HOMA-IR and that acupuncture could significantly improve HOMA-IR and the level of fasting glucose in patients with PCOS, which confirms previous reports [27, 28]. It is also reported that acupuncture can decrease BMI and WHR [28, 42, 43]. FINS, 2hFPG, and 2hFINS were reduced in the acupuncture groups, but these differences were not significantly different. The results of the lipid profile were reported in a few studies, and we found that acupuncture significantly improved TG levels, while the differences in TC, LDL-C, and HDL-C were not significant. Acupuncture seems to be associated with a few adverse events, and the reported adverse events, such as bleeding, were mild and transient, demonstrating that acupuncture is safe and reliable. Thus, we conclude that acupuncture, compared with standard therapy, is more effective and safer in improving glucose metabolism and insulin sensitivity in patients with PCOS.

### 4.2. Limitations

First, this meta-analysis only included clinical studies comparing acupuncture alone versus placebo (placebo or sham acupuncture) or standard therapy. Studies on other combined therapies were not included because the combined therapies are more complex than monotherapy. Indeed, if acupuncture alone can achieve a curative effect without significant cost or time, then additional therapies might not be necessary. Second, there are certain heterogeneities among these studies. On the one hand, PCOS itself is heterogeneous by nature in terms of its clinical and biochemical features, and different distributions of ethnicity and age contribute to different manifestations of PCOS [44]. On the other hand, the disease severity and the dosages of metformin differed among the studies. Finally, some of the included studies did not describe the specific randomization method or did not adopt blinding. Therefore, these studies have just an average methodological quality.

### 4.3. Implications for Clinical Practice and Further Research

The results of this meta-analysis suggest that acupuncture alone has better efficacy than control interventions. Because traditional Chinese medicine departments have been established in most maternity hospitals and general hospitals in China, it is easy and convenient to apply such treatments. As a safe and simple therapy, acupuncture might be an alternative or a good adjunct therapy for PCOS, especially for overweight patients and patients with IR. However, we have no information on how long the effect of acupuncture might last. Thus, more long-term follow-up studies are needed to examine the effectiveness of acupuncture in improving insulin resistance and depressing BMI and WHR and to assess the sustainability of their effects.

Several meta-analyses have been performed regarding the effectiveness of acupuncture for treating PCOS, specifically focusing on rates of ovulation, pregnancy, and live birth [45–47], but articles about how acupuncture affects metabolic-related indexes in patients with PCOS, particularly lipid profiles, are lacking.

## 5. Conclusion

There is insufficient evidence to support that acupuncture can improve lipid profiles except for TG levels. However, this review of 10 RCTs shows that acupuncture could improve BMI and WHR as well as HOMA-IR in patients with PCOS. The included studies are inconclusive because of their moderate level of evidence, and further large-scale, long-term RCTs with rigorous methodological standards are still warranted.

## Figures and Tables

**Table 1:** Features of the included studies.

Study ID	Sample size	Course of PCOS	Participant characteristics	Age	Treatment versus control	Outcome
Stener-Victorin, 2009	15			T: 29.9 ± 4.5 C: 30.4 ± 5.5	Electroacupuncture group: the needles were inserted into acupoints including CV3, RN6, bilateral ST29, SP6, and LI4 (or PC6 alternatively every second time) and retained for 30 min. The procedure was administered two times a week for 2 weeks, once a week for 6 weeks, and then once every second week for 8 weeks, giving a total of 14 treatments over 16 weeks. Control group: this group was given information about the importance of physical activity and a healthy diet.	BMI, WHR, FGS, FPG, FINS, HOMA-IR, TC, TG, LDL-C, HDL-C
Zheng, 2012	86	T: 3.5 ± 2.8 C: 3.6 ± 2.2	Obesity-type PCOS	T: 26.5 ± 3.0 C: 24.9 ± 4.9	Acupuncture group: the needles were inserted into acupoints including RN4, RN6, RN10, CV12, ST21, ST25, and ST28 and retained for 30 min. The procedure was administered twice a week for 6 months. Control group: metformin was taken with food for 6 months. In the first week of the study, patients received 250 mg three times daily; thereafter, the metformin dose was 500 mg three times daily.	BMI, WHR, FGS, FGP, FINS, 2hFPG, 2hFINS, HOMA-IR, TC, TG, LDL-C, HDL-C
Yao, 2018	97	T: 3.1 ± 0.9 C: 3.2 ± 0.8	Obesity-type PCOS	T: 27.8 ± 4.8 C: 28.2 ± 4.5	Acupuncture group: the needles were inserted into acupoints including RN17, CV12, RN4, bilateral BL18, ST25, RN19, ST36, LR14, SP6, and LR3 and retained for 30 min. The procedure was administered three times a week for 6 months. Control group: metformin was taken at 500 mg three times daily with food for 6 months.	BMI, WHR, FGS, 2hFPG, HOMA-IR
Kong, 2015	55		Obesity-type PCOS	T: 28.1 ± 3.8 C: 27.8 ± 3.4	Electroacupuncture group: the needles were inserted into acupoints including DU20, CV3, RN6, bilateral LI4, SP6, ST29, and SP9 and retained for 30 min. The needles were then inserted into acupoints including DU20, CV3, bilateral ST29, PC6, SP6, ST25, and LR3 and retained for 30 min. The procedure was performed two or three times a week for a total of 32 sessions. Control group: Sham acupuncture was used, and virtual electroacupuncture was used at the acupoint.	BMI, WHR, FGP, FINS, HOMA-IR, TC, TG, LDL-C, HDL-C
Peng, 2017	100	T: 2.5 ± 0.8 C: 2.4 ± 0.9		T: 28.6 ± 3.8 C: 28.8 ± 3.3	Acupuncture group: the needles were inserted into acupoints including SP6, ST36, ST40, and ST25 and retained for 30 min. The procedure was administered three times a week for 3 months. Control group: Sham acupuncture was administered three times a week for 3 months.	BMI, WHR
Li, 2014	100				Acupuncture group: the needles were inserted into acupoints including CV3, RN4, bilateral ST36, SP6, KI7, and RN6 and retained for 30 min. The procedure was performed every day except during the menstrual period. Control group: both sham acupuncture and metformin were used. Metformin was taken as a single tablet with food three times daily for 6 months.	BMI, WHR, FGS, FGP, FINS, HOMA-IR, TC, TG, LDL-C, HDL-C
Cai, 2016	50		Obesity-type PCOS		Acupuncture group: the needles were inserted into acupoints including CV12, ST21, ST25, GB26, RN6, RN4, ST28, SP10, ST34, ST36, ST37, and SP6 and retained for 30 min. The procedure was performed three times a week for 3 months. Control group: metformin was taken at 500 mg three times daily with food for 6 months.	BMI, WHR, FGP, FINS, HOMA-IR
Lai, 2012	120	T: 2.5 ± 0.6 C: 2.5 ± 0.7		T: 26.7 ± 2.7 C: 26.5 ± 2.7	Acupuncture group: the needles were inserted into acupoints including CV12, RN10, RN6, RN4, ST25, and ST28 and retained for 30 min. The procedure was performed once every 3 days for 4 months. Control group: metformin was taken at 500 mg three times daily with food for 4 months.	BMI, WHR, FGP, FINS, 2hFPG, 2hFINS, HOMA-IR
Guo, 2014	38		PCOS-IR	T: 27.8 ± 3.2 C: 29.3 ± 2.9	Electropuncture group: the needles were inserted into acupoints including CV3, RN6, DU20, bilateral ST29, SP6, SP9, and LI4 and retained for 30 min. The needles were then inserted into acupoints including CV3, RN6, DU20, bilateral ST25, ST29, SP6, LR3, and PC6 and retained for 30 min. The procedure was performed once every 2 days for 2 months. Control group: Sham acupuncture was used and retained for 30 min. The procedure was performed once every 2 days for 2 months.	BMI, WHR, FGS, FGP, FINS, HOMA-IR, TC, TG, LDL-C, HDL-C
Gu, 2019	76	T: 4.6 ± 3.6 C: 4.3 ± 3.3	PCOS-IR	T: 27.0 ± 4.5 C: 28.6 ± 4.0	Electroacupuncture group: the needles were inserted into acupoints including CV3, RN6, DU20, bilateral ST29, SP6, SP9, and LI4 and retained for 30 min. The needles were then inserted into acupoints including CV3, RN6, DU20, bilateral ST25, ST29, SP6, LR3, and PC6, and retained for 30 min. The procedure was performed twice a week for a total of 32 sessions. Control group: Sham acupuncture was used and retained for 30 min. The procedure was performed twice a week for a total of 32 sessions.	BMI, WHR, FGP, 2hFPG, FINS, 2hFINS, HOMA-IR, TC, TG, LDL-C, HDL-C

BMI: body mass index; WHR: waist-to-hip ratio; FGS: Ferriman–Gallwey score; FPG: fasting plasma glucose; 2hFPG: 2 h fasting plasma glucose; FINS: fasting insulin; 2hFINS: 2 h fasting insulin; HOMA-IR: homeostasis model assessment of insulin resistance; TC: serum total cholesterol; TG: triglyceride; LDL-C: low-density lipoprotein cholesterol; HDL-C: high-density lipoprotein cholesterol.
